# Antigenicity Preservation Is Related to Tissue Characteristics and the Post-Mortem Interval: Immunohistochemical Study and Literature Review

**DOI:** 10.3390/healthcare10081495

**Published:** 2022-08-08

**Authors:** Silvestro Mauriello, Michele Treglia, Margherita Pallocci, Rita Bonfiglio, Erica Giacobbi, Pierluigi Passalacqua, Andrea Cammarano, Cristian D’Ovidio, Luigi Tonino Marsella, Manuel Scimeca

**Affiliations:** 1 Department of Biomedicine and Prevention, University of Rome “Tor Vergata”, Via Montpellier 1, 00133 Rome, Italy; 2 Department of Experimental Medicine, University of Rome “Tor Vergata”, Via Montpellier 1, 00133 Rome, Italy; 3 Department of Medicine and Aging Sciences, University of Chieti-Pescara “G. D’Annunzio”, Section of Legal Medicine, 66100 Chieti, Italy

**Keywords:** immunohistochemistry, protein degradation, post-mortem interval, time since death, forensic pathology, autopsy

## Abstract

The main aim of this study was to investigate the post-mortem proteolytic degradation process of selected tissue antigens and correlate it to the post-mortem interval. During the autopsy of 12 cadavers (time interval ranging 1 day–2 years after death) samples of skin, liver, kidney, and spleen were collected. All samples were formalin-fixed and paraffin-embedded. Four µm paraffin sections were used for hematoxylin–eosin staining and immunohistochemical analysis (Ki67, Vimentin, Pan cytokeratin, and CD20). Data reported here show that immunohistochemical reactivity preservation was related to the characteristics of the tissues. In particular, the most resistant tissue was the skin, where the autolysis phenomena were not appreciable before 5 days. On the contrary, the liver and the spleen underwent early autolysis, while the kidney displayed an early autolysis of the tubules and a late one of the glomeruli. As concerns specific antigens, immunoreactivity was lost earliest for nuclear antigens as compared to cytoplasmic ones. In conclusion, our results demonstrate that immunohistochemical detection of specific antigens may be useful in estimating the post-mortem interval, especially when we need to know whether the post-mortem interval is a few days or more than 7–10 days.

## 1. Introduction

The identification of the post-mortem interval (PMI) in cadavers involved in judicial investigations is of particular relevance in the daily practice of forensic pathologists. The elements on which the medico–legal expert must base judgement are given by both the objectification of the cadaveric phenomena and tanatochronology science. The study of these parameters contributes to the construction of a time-dependent curve whose characteristics (e.g., slope) and initial point (time of death) can be influenced by internal, external, ante- or post-mortem factors [1]. The methods used in PMI estimation are based on the evaluation of post-mortem changes due to physical (cooling and hypostasis), metabolic (concentrations of particular metabolites, enzymatic activities), autolytic (loss of cell membrane selectivity and morphological changes), physiological and chemical (supravital reactivity, rigor mortis), and bacterial (putrefaction) processes [1]. The methods to estimate the time since death are not only different in nature but also have a widely differing scientific value concerning, for example, the validation of the method [2]. In addition, it has been shown that the farther away from the time of death, the greater the chance that the PMI estimate will be more difficult and therefore less reliable [3]. The lack of reliable methods is particularly evident, especially for the determination of PMI in the intermediate phase (between 24 h and about 7 days post-mortem). [4]. Thus, numerous additional approaches have been proposed in recent years to overcome the deficits and gaps in the applied spectrum of conventional PMI delimitation methods. The introduction of the line of research on protein degradation in the estimation of the post-mortem interval is quite recent. The first study focused on the death time detection according to the evaluation of protein degradation was published in 1999 [5]. After this interesting study, a growing body of evidence highlighted the potential of protein degradation analysis as PMI method. The main analysis used to the study of protein degradation after death are immunohistochemistry and Western blot. Immunohistochemistry is based on the specificity of primary antibodies to bind antigens localized in the subcellular compartment, such as the membrane, cytoplasm, and nucleus. It has been shown that after death a process of modification of the tertiary structure of proteins occurs that varies from protein to protein and that, in the long-term, leads to the complete degradation of the antigen. The post-mortem proteolytic process is related to different mechanisms; some authors suggest that an important role in this sense is played by calpains, enzymes that are activated in all those situations in which there is an increase in intracellular calcium concentrations [6,7]. In addition, the post-mortem degradation of proteins is accelerated by enzymes released by bacterial cells involved in putrefaction and, in more advanced stages, also by enzymes released by necrophagous insects [5].

The use of immunohistochemical investigations in the forensic field is related to the possibility offered by this technique to study the conservation of tissue antigens and to determine the PMI (assuming that there is a physiological depletion) and time-dependence of protein markers. Therefore, the identification and standardization of protein degradation analysis can include the investigations aimed at establishing the time of death, especially those in thanatological situations, such as putrefaction, where the classical macroscopic and microscopic analysis show several limitations.

Starting from these considerations, the main aim of this study was to investigate the post-mortem proteolytic degradation process of selected tissue antigens and correlate it to the PMI.

## 2. Materials and Methods

### 2.1. Case Selection

During autopsy of 12 cadavers, performed with a time interval ranging from 1 day to 2 years after death, samples of skin, liver, kidney, and spleen were taken. All skin samples were taken at the abdominal level, as close as possible to the incisura.

In order to reduce, as much as possible, inaccuracy, only cases with well-known time since death and anamnestic information (sex, age, and cause of death) were collected. Cases with macroscopic pathologies of the liver, spleen, and kidney were excluded. Bodies found both indoors and outdoors were included in the study. External factors such as temperature, weather, and clothing varied widely from case to case.

Autoptic examinations were performed 1 day after death in 4/12 cases (3 men and 1 woman; mean age 33.8 ± 6.5 years), 3 days after death in 2/12 cases (1 man and 1 woman; mean age 29.25 ± 7.32 years), 5 days after death in 2/12 cases (1 man and 1 woman; mean age 31.12 ± 4.99 years), 15 days after death in 2/12 cases (1 man and 1 woman; mean age 39.75 ± 7.85 years) and 2 years after death in 2/12 cases (1 man and 1 woman; mean age 41.2 ± 3.38 years). Cause of death was “violent death” for all cases.

### 2.2. Histology

After the autopsy, all the samples were fixed in formalin for 24 h and embedded in paraffin wax. From each block, serial sections were taken, subjected to hematoxylin–eosin staining and immunohistochemical studies.

### 2.3. Immunohistochemistry

The immunoreactivity of the samples was investigated by performing immunohistochemical reaction of the following antigens: vimentin, ki67, cytokeratin (CK), and CD20. Briefly, sections were pre-treated using heat mediated antigen retrieval with sodium citrate buffer (pH6) or EDTA citrate (pH 7.8) for 30 min at 95 °C. The section was then incubated with primary monoclonal antibodies listed in Table 1. Pan-CK antibody was used for liver, kidney, and skin tissues. Vimentin and Ki67 antibodies were used for spleen, liver, kidney, and skin tissues. CD20 antibody was used for the spleen.

Washings were performed using PBS/Tween20 pH 7.6. Reactions were detected using an HRP-conjugated compact polymer system HRP-DAB kit (UCS diagnostics, Rome, Italy).

The following antigens were used as markers for immunohistochemical analysis:

(a) Vimentin

Vimentin, derived from latin word vimentum (flexible twig bunch), is an intracellular protein and is a type III intermediate filament protein. This protein is expressed in fibroblasts, endothelial cells, and lymphocytes [8] in which they polymerize to form the basis of the cytoskeleton, allowing for the maintenance of cellular structure in addition to contributing to cell signaling and proliferation [9]. This protein is found in various non-epithelial cells, especially mesenchymal cells [10], and stains melanocyte cells and dermal vessels. Structurally, the vimentin monomer contains a non-helical amino head, the central structure of the alpha helix domain, and a carboxyl tail. Subsequently, the monomers combine to form dimers that represent the basic subunits of vimentin [11].

(b) Ki 67

Ki-67 is a nuclear DNA-binding protein constitutively expressed in cycling mammalian cells [12]. It exists in two human isoforms with a maximum expression in G2 phase or during mitosis [12]. It prevents chromosomes from collapsing into a single chromatin mass by forming a steric and electrostatic charge barrier: the protein has a high net electrical charge and act as a surfactant, dispersing chromosomes and enabling independent chromosome motility [13]. Ki-67 represents a useful marker of cell proliferation [14].

(c) Cytokeratin

CKs are a heterogeneous group of proteins that contribute to the structure of intermediate filament that function as the supporting cytoskeleton in the epithelial cells [15]. The type I CKs consist of acidic proteins, arranged in pairs of heterotypic keratin chains, and the type II are basic or neutral proteins which are arranged in pairs of heterotypic keratin chains co-expressed during differentiation of simple and stratified epithelial tissues [16]. It has been demonstrated that keratin expression can be found in subsets of several types of mesenchymal cells [17].

(d) CD20

CD20 is a B-cell marker expressed starting from late pre-B lymphocytes [18]; its expression is lost in terminally differentiated plasmablasts and plasma cells. It is a nonglycosylated 33–37 kDa protein member of the MS4A family. Structurally it consists of four hydrophobic transmembrane domains, one intracellular and two extracellular domains [18]. The extracellular portion of CD20 is 44 amino acids in length and provides the docking site for anti-CD20 MAbs binding. CD20 expression is initiated at the pre-B cell stage of development and remains present until terminal differentiation into a plasma cell [19].

Positive controls were performed by using multi-organ tissue micro arrays (TMA). Negative controls were performed without using the primary antibody.

## 3. Results

### 3.1. Morphological Analysis

Hematoxylin–eosin staining allowed us to evaluate the morphological characteristics and conservation status of all tissues.

#### 3.1.1. Skin

**Figure 1:**
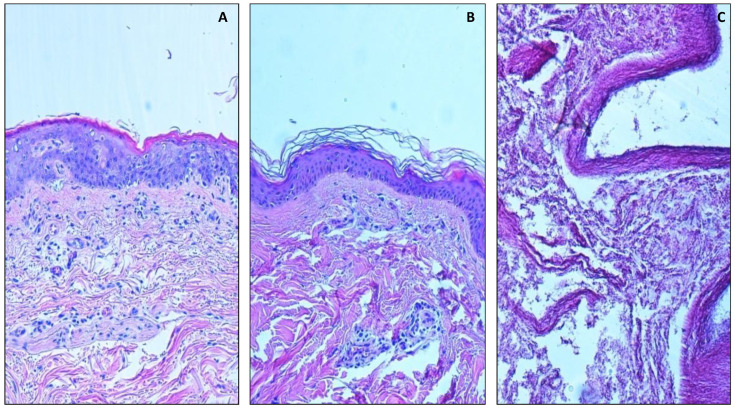
Morphological analysis of skin. (**A**,**B**) Image shows well preserved skin 15 days after death, 10×. (**C**) Disgregated skin 2 years after death, 10×.

The skin showed a good preservation of its structure in all samples taken up to 15 days after death (Figure 1A,B). The different layers of the epidermis and the annexal structures of the dermis were well recognizable, while the morphological structure of the skin was no longer recognizable in samples taken two years after death (Figure 1C).

#### 3.1.2. Kidney

**Figure 2:**
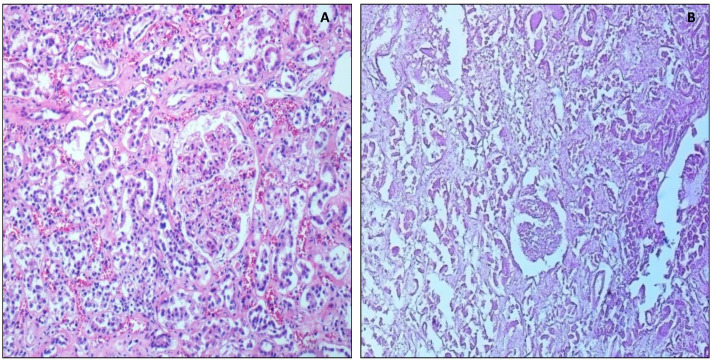
Morphological analysis of kidney. (**A**) Image displays well preserved glomeruli and tubules 1 day after death, 10×. (**B**) Disgregated glomeruli and tubules 2 years after death, 10×.

Kidneys taken 1 day after death displayed a perfectly recognizable structure of the parenchyma, the glomeruli, and tubules (Figure 2). At 3 days, there was a progressive autolysis of tubular cells with partial visualization of isolated tubular structures in contrast to the glomeruli, which were still preserved (Figure 2A). At 5 days, it was possible to observe partial autolysis of the glomerular cells. Lastly, at 15 days no cellular structures of the tubules and glomeruli were recognizable (Figure 2B).

#### 3.1.3. Liver

**Figure 3:**
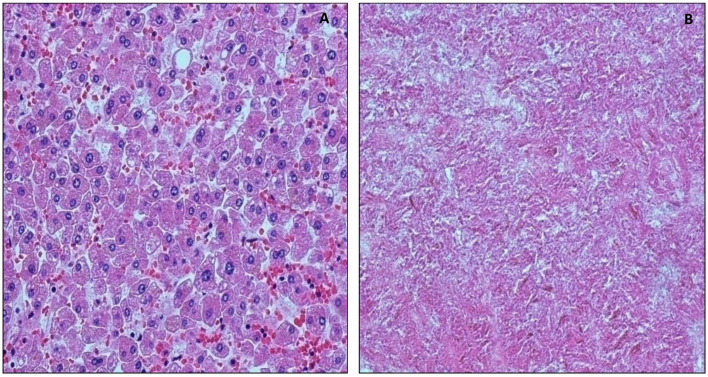
Morphological analysis of liver. (**A**) Image displays well preserved glomeruli and tubules 1 day after death, 20×. (**B**) Disgregated glomeruli and tubules 2 years after death, 10×.

The structure of the liver was well preserved in the sample taken at one day after death, in which the hepatocytes were clearly identifiable. Rare focal autolytic phenomena of the hepatocytes were observed in specimens taken 3 days after death (Figure 3A). Autolysis phenomena were slightly increased in the 5-day specimen collection, while at 15 days, tissue autolysis was completed (Figure 3B).

#### 3.1.4. Spleen

**Figure 4:**
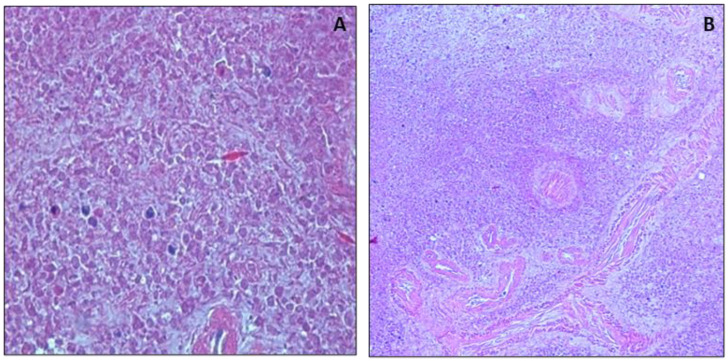
Morphological analysis of spleen. (**A**) Image displays well preserved spleen 5 days after death, 10×. (**B**) Image shows altered spleen parenchyma 15 days after death, 10×.

The morphostructure of the spleen in samples taken one day after death was well preserved, and cellular components in the red pulp and white pulp were clearly visible. In the 3-day sample, the lymphatic structures in the white pulp were still clearly visible and preserved, while a progressive autolysis of the red pulp cells was observed. Progressive autolysis of white pulp lymphocytes was also observed in specimens taken 5 days after death (Figure 4).

### 3.2. Immunohistochemical Analysis

#### 3.2.1. Anti-Vimentin Antibody

**Figure 5:**
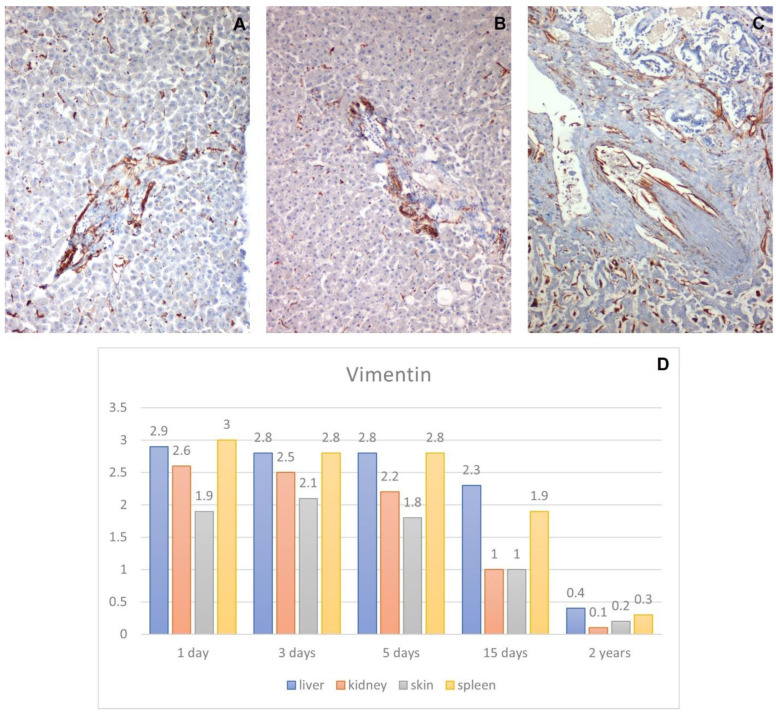
Immunohistochemical analysis of vimentin. (**A**) high vimentin expression in liver taken 1 day after death, 10×. (**B**) High vimentin expression in liver taken 3 days after death, 10×; (**C**) high vimentin expression in liver taken 15 days after death, 10×. (**D**) Graph shows the intensity of vimentin immunostaining (score 0–3).

**Figure 6:**
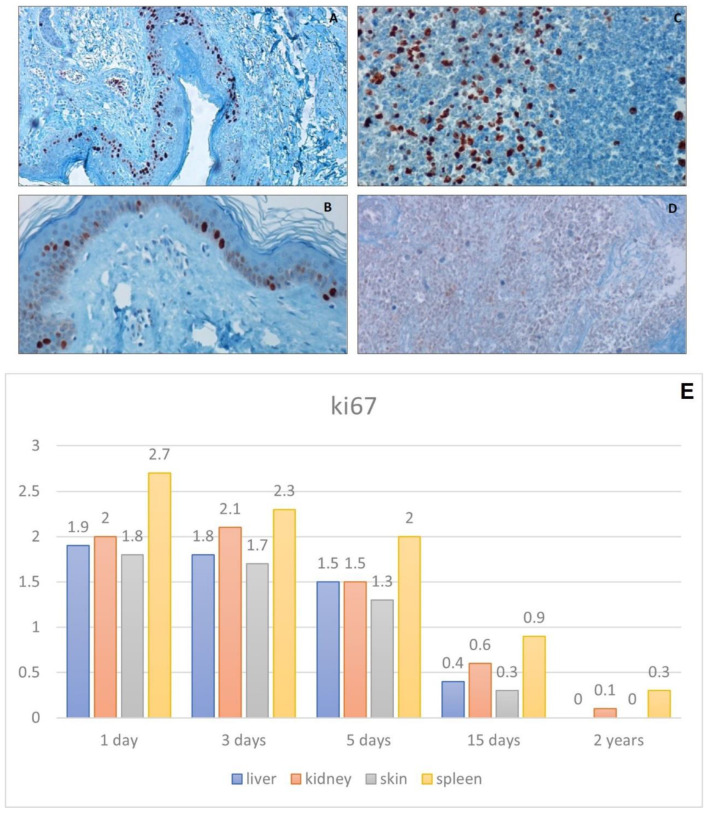
Immunohistochemical analysis of ki 67. (**A**) high nuclear Ki67 expression in skin taken 1 day after death, 10×. (**B**) Reduction of nuclear Ki67 expression in skin taken 5 day after death, 10×. (**C**) Ki67 nuclear expression in spleen lymphocytes (3 days after death). 20× (**D**) No Ki67 nuclear staining in a spleen sample taken 5 days after death, 10×. (**E**) Graph shows the intensity of ki67 immunostaining (score 0–3).

Staining was positive and specific in all examined samples up to day 5 after death, although it slightly lost intensity. In the liver, the small portal vessels were stained (Figure 5A). In the kidney, the cytoplasm of some tubule cells and some glomerular endothelial cells were stained for the vimentin and were specific and intense until the third day after death (Figure 6B). Starting from the fifth day after death, only some glomerular endothelia were stained, while the tubules were completely negative. In the two-year sample, the vimentin signal was completely lost.

In the spleen, vimentin stains the endothelium of the chordae of the red pulp, and the staining was specific and abundant at day one, while it was completely negative on day 3–5 due to the presence of autolytic processes (Figure 6C).

#### 3.2.2. Anti-Ki67 Antibody

The skin showed a strong staining in the one-day specimens (Figure 6A); with the increase of the PMI, a decrease in the percentage of positive cells was appreciated. At the same time, specimens showed slight aspecific signal (background) (Figure 6B).

In the spleen, the positivity was closely related to the degree of autolysis. The ki67 stain maintained a good ratio until the third day after death, where non-specific signals were observed (Figure 6C,D).

#### 3.2.3. Anti-Pan-Cytokeratin Antibody

**Figure 7:**
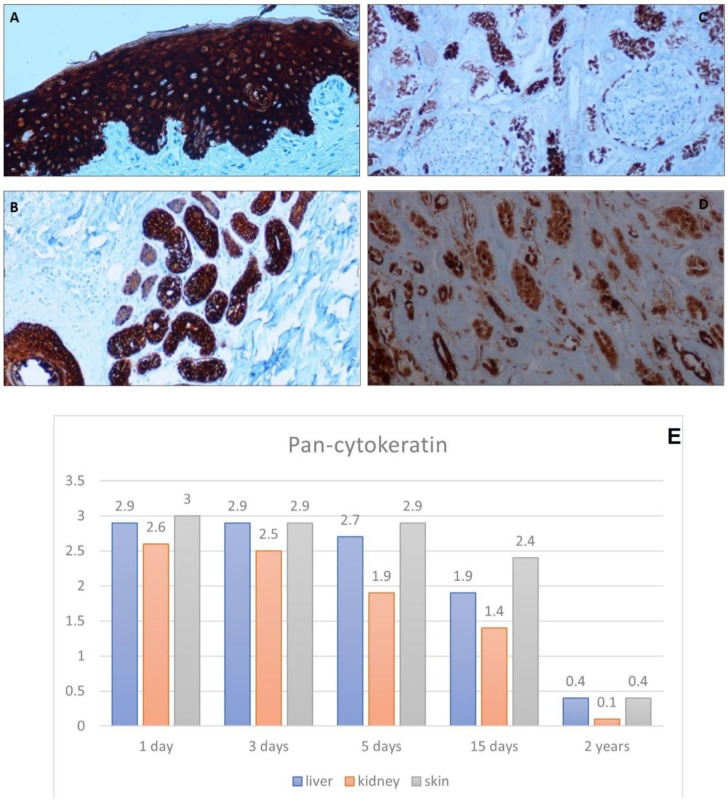
Immunohistochemical analysis of pan-Cytokeratin. (**A**) very high staining of pan cytokeratin in skin tissue taken 3 days after death, 20×. (**B**) Subcutaneous tissue taken 3 days after death displays high pan-cytokeratin expression, 10×. (**C**) High pan cytokeratin expression in a kidney taken 1 day after death, 10×. (**D**) Aspecific pan cytokeratin staining in a kidney tissue 5 days after death, 20×. (**E**) Graph shows the intensity of pan-cytokeratin immunostaining (score 0–3).

In the skin, CK reactivity was perfectly maintained both on the epidermis and on the adnexal structures in all the samplings taken up to 3 days (Figure 7A,B). Keratin remained expressed with a specific localization in the epidermis with a concomitant partial diffusion in the other tissues in the two-year sampling.

In the liver, hepatocyte staining was maintained in all preparations, although as a result of autolytic phenomena, positivity tended to become less specific. In the kidney, the antibody stained the tubules and the parietal cells of the glomerulus as well as the Bowmann’s capsule. The positivity was maintained in the ducts that do not present autolytic phenomena even in 3–5 days but became aspecific by also staining the nuclei of the cells in autolysis (Figure 7C,D).

#### 3.2.4. Anti-CD20 Antibody

**Figure 8:**
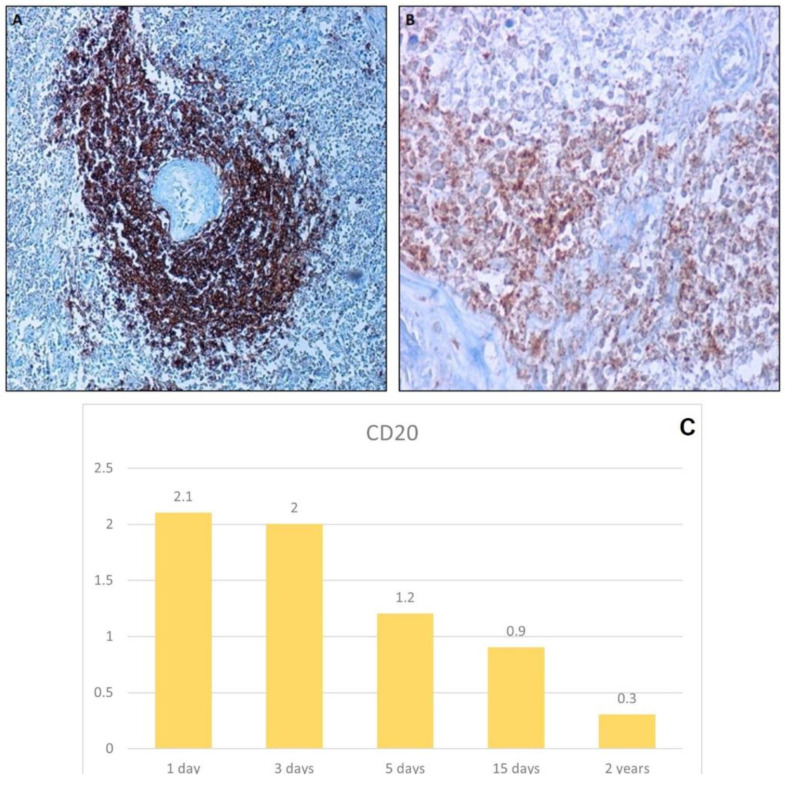
Immunohistochemical analysis of CD20. (**A**) CD20 positive cells in a spleen taken 2 days after death, 10×; (**B**) the CD20 staining became faint and less specific 5 days after death, 40×. (**C**) Graph shows the intensity of CD20 immunostaining in the spleen (score 0–3).

To evaluate the immunoreactivity of CD20, a typical membrane protein that stains B-lymphocytes, splenic tissue was used since in it numerous lymphoid follicles are normally present. The reactivity was intense and specific in the one- to two-day samples (Figure 8), whereas in the five-day sample, it became less specific (Figure 8).

Table 2, Table 3, Table 4 and Table 5 summarize the results of immunohistochemistry in the tissues analyzed.

## 4. Discussion

Immunohistochemistry represents a promising application in the forensic and medico–legal field, and its use can be exploited, together with other methods, to reach multiple objectives. In fact, immunohistochemistry is currently used for the dating of lesions and to support the differential diagnostic workflow between vital and post-fatal lesions [20,21,22]. Some authors have instead investigated the feasibility of employing immunohistochemistry in the diagnosis of death, achieving in some cases promising results [23,24,25]. Since the 1990s, immunohistochemical studies have also been applied in the estimation of the PMI. In this field of application, along with entomological studies and high-resolution 1H magnetic resonance tomography, immunohistochemical methods are particularly suited for delimitating long PMI [3,26,27,28,29,30]. A brief review of the literature revealed that only a few studies have been published based on the analysis of post-mortem protein degradation by immunohistochemistry to estimate the time of death (Table 6).

In six studies, tissues from internal organs such as thyroid, pancreas, and brain were used [3,27,28,29,30,31], while in the other two [26,27,28,29,30,31,32], the analysis was performed on samples of gingival and skin tissue. The main protein markers investigated were insulin and glucagon for pancreatic tissue, calcitonin and thyroglobulin for thyroid, collagen type I and III for gingival tissue, GFAP for brain, and CEA, S-100 protein, CK and smooth muscle actin for sweat glands. In all the examined cases, the PMI was known before the autopsy, and the analyzed samples were heterogeneous with regard to (a) the environmental conditions in which the corpses were found, (b) the body structure, and (c) the causes of death. Almost all mentioned studies show a progressive reduction of the positivity of the specific marker until the complete negativity as the PMI interval increases, except for the study conducted by Cingolani et al. [26]. This could be explained by considering the short PMI used by the authors.

Regarding thyroglobulin, used in two studies as a specific marker for thyroid tissue, the results showed a significant variability; in particular, according to Wehner et al. [30], all analyzed cases within the fifth day after death were positive, with progressive reduction of the number of positive cases until the twelfth day. A complete negativization of cases was observed starting from the 13th day. In the study conducted by Ortmann et al., [31] some tissues showed negativity for thyroglobulin already from the second day, with progressive reduction of the number of positive cases until the eighth day after death. Glucagon exhibited similar patterns, with positivity maintained in most cases up to 6–8 days and complete negativization from day 12–13 onwards. Among all studied markers, insulin showed the longest persistence, with positivity of all cases maintained until day 12 and complete negativity of samples only starting from day 30 after death. Our results show that the immunoreactivity for nuclear antigens is lost earlier as compared to the cytoplasmic ones. In addition, we observed that the preservation of immunohistochemical reactivity is related to the intrinsic characteristics of the analyzed tissues and their different ability to withstand autolysis at the same PMI.

The most resistant tissue seems to be the skin, since autolysis phenomena are not appreciable before 5 days’ postmortem; the liver, but especially the spleen, undergo early autolysis, while the kidney presents a variable autolysis degree, showing an early alteration of the tubules. In this regard, our results confirmed the usefulness of vimentin and pan-CK staining for the assessment of tissue preservation status. The vimentin tends to become negative only when marked autolysis is occurring, such as in the spleen after the third day or in renal tubules on the fifth day, while it remains positive, even if with a slight decrease in intensity, in the skin. The results of staining with antibodies against pan-CK, a cytoplasmic marker, are like those obtained with vimentin, showing, however, a greater capacity for preservation. In fact, whereas vimentin was completely negative in skin samples at two years, CK staining persisted in the epidermis. Reactivity for antibodies against membrane antigens, such as CD20, demonstrates rapid degradation as observed in the spleen where the positivity becomes nonspecific or negative already 2 days after death.

Preservation of antigenic determinants is one of the most important methodological problems of immunohistochemical studies [33]. Fixation preserves cell and tissue morphology by preventing antigen destruction; inappropriate fixation can partially destroy antigenic determinants or alter their structure to such an extent that they are unrecognizable to the antibody. Conversely, an unfixed antigen may disappear altogether or may diffuse from the site of synthesis into the surrounding tissue.

Factors such as cold ischemia time in surgical settings, PMI, fixation time, paraffin, storage time in paraffin, storage temperature, age of cut sections, antigen retrieval technique, and detection systems have been reported to influence the outcome of immunohistochemistry [3]. Furthermore, post-mortem tissues are also subjected to specific variables such as the agonal state; this can influence the tissue characteristics and immunoreactivity by pH changes [34]. In addition, the strong influence that climatic and environmental factors seem to have on the reliability of immunohistochemical results should be kept in mind. In accordance with the observations of some authors, at the same PMI, markedly more negative immunoreactions are seen in the warmer months than in the cooler months [30]. These differences are obviously due to the influence that the temperature has on the proteolytic and autolytic processes. It is therefore necessary to take this into account for all mentioned variables that could significantly alter the tissue immunoreactivity.

## 5. Conclusions

The results of this preliminary study suggest that immunohistochemical detection of specific antigens may be useful in estimating the PMI. Our findings demonstrated that the study of post-mortem antigenic degradation, especially for vimentin, pan-CK and ki67, could be promising when we need to know whether the PMI is a few days or more than 7–10 days. Therefore, further studies on large sample sizes are necessary to investigate how the variables related to causes of death, environmental conditions in which the corpse remains before being found, timing of retrievals with respect to the time of death, and mode of preservation and fixation of tissues may affect the protein degradation. To make this method reliable for forensic practice, it would also be desirable to carry out studies aimed at analyzing the behavior of different antigens or to evaluate the simultaneous use of multiple parameters for improving the accuracy of PMI estimation. In this context, cadavers obtained from cadaveric donation programs for scientific research purposes that are becoming established worldwide could profitably be used as a study sample [35].

## Figures and Tables

**Table 1:** List of primary antibodies.

Antibody	Characteristics	Dilution	Retrieval
anti-PanCytokeratin	mouse monoclonal clone [AE1/AE3]; Ventana, Tucson, AZ, USA	Pre-diluted	EDTA citrate pH 7.8
anti-Vimentin	mouse monoclonal clone V9; Ventana, Tucson, AZ, USA	Pre-diluted	EDTA citrate pH 7.8
anti-Ki67	rabbit monoclonal clone (30-9); Ventana, Tucson, AZ, USA	Pre-diluted	Citrate pH 6.0
anti-CD20	mouse monoclonal clone L26; Ventana, Tucson, AZ, USA	1:100	EDTA citrate pH 7.8

**Table 2:** Immunohistochemical evaluation of cytokeratin, ki67, and vimentin in skin tissues.

Post Mortem Interval	Hematoxylin—Eosin Staining	Anti-Pan-CK Staining	Anti-Ki 67 Staining	Anti-Vimentin Staining
1 day	Preservation of epidermis and cutaneous annex structure	Positive and specific staining in all samples	Intense staining	Positive and specific staining in all samples
3 days	Preservation of epidermis and cutaneous annex structure	Positive and specific staining in all samples	Intense staining	Positive and specific staining in all samples
5 days	Preservation of epidermis and cutaneous annexes structure	Positive and specific staining in all preparations with reduction in intensity	Quantitative decrease of positive cells	Positive and specific staining in all preparations with reduction in intensity
15 days	Preservation of epidermis and cutaneous annexes structure	Positive and specific staining in all preparations with reduction in intensity	Lack of positive cells. Non-specific background staining for increased cytoplasmic staining of negative cells	Negative staining
2 years	Negative staining	Negative staining	Negative staining	Negative staining

**Table 3:** Immunohistochemical evaluation of cytokeratin and vimentin in kidney tissues.

Post Mortem Interval	Hematoxylin—Eosin Staining	Anti-Pan-CK Staining	Anti-Vimentin Staining
1 day	Parenchymal structure clearly detectable with preservation of both glomeruli and tubules	Specific and intense positivity	Specific and intense positivity
3 days	Progressive autolysis of tubular cells with evidence of only isolated tubular structures. Glomeruli still preserved	Specific and intense positivity	Specific and intense positivity
5 days	Parcellular autolysis of glomerular cells	Decreased positivity for autolytic phenomena	Positivity detectable only at the level of some glomerular endothelia while tubules in autolysis are negative
15 days	No longer detectable any cellular structure of the tubules and glomeruli	Non-specific staining	Negative staining
2 years	Negative staining	Negative staining	Negative staining

**Table 4:** Immunohistochemical evaluation of ki67, vimentin, and CD20 in spleen tissues.

Post Mortem Interval	Hematoxylin—Eosin Staining	Anti Ki-67 Staining	Anti-Vimentin Staining	Anti-CD20 Staining
1 day	Structure of the parenchyma preserved, cellular components are detectable in the red pulp and in the white pulp	Well stained with structure retention	Positive and specific staining in all samples	Positive and specific in all samples
3 days	Lymphatic structures in the white pulp are still well preserved and visible, while a progressive autolysis of red pulp cells is observed	Well stained with structure retention	Negative staining	Positive and specific in all samples
5 days	Progressive autolysis of white pulp lymphocytes appearing as cellular shadows	Rare positive cells	Negative staining	Decreased positivity for autolytic phenomena
15 days	Complete tissue autolysis	Complete tissue autolysis	Negative staining	Non-specific staining
2 years	Negative staining	Negative staining	Negative staining	Negative staining

**Table 5:** Immunohistochemical evaluation of cytokeratin and vimentin in liver tissues.

Post Mortem Interval	Hematoxylin—Eosin Staining	Anti-Pan-CK Staining	Anti-Vimentin Staining
1 day	Well-preserved structure with clear recognition of hepatocytes	Positive and specific staining in all samples	Positive and specific staining in all samples
3 days	Tissue morphology mostly maintained despite foci of hepatocyte autolysis beginning to appear	Positive and specific staining in all samples	Positive and specific staining in all samples
5 days	Enhanced autolysis phenomena	Positive and specific staining in all preparations with reduction in intensity	Positive and specific staining in all preparations with reduction in intensity
15 days	Complete tissue autolysis	Non-specific staining	Negative staining
2 years	Negative staining	Negative staining	Negative staining

**Table 6:** Summary overview of study characteristics.

Author and Year	Tissue Sample	Marker	Sample Size and Study Group
Cingolani M. et al., 1994 [26]	Skin (sweat glands)	S-100 protein, carcinoembryonic antigen (CEA), cytokeratin, actin smooth muscle (ASM)	n = 29 corpses with known time since death; samples were taken at intervals of 3, 6, 9 and 12 h after death
Wehner F. et al., 1999 [27]	Pancreas	Insulin	n = 128 corpses with known time since death (between 1 day and 445 days)
Wehner F. et al., 2000 [28]	Thyroid	Thyreoglobulin	n = 147 corpses with known time since death (between 1 day and 21 days)
Wehner F. et al., 2001 [29]	Thyroid	Calcitonin	n = 136 corpses with known time since death (between 1 day and 21 days)
Wehner F. et al., 2001 [3]	Pancreas	Glucagon	n = 214 corpses with known time since death (between 1 day and 21 days)
Wehner F. et al., 2006 [30]	Pancreas and brain (frontal cortex)	Glial fibrillary acidic protein (GFAP) and somatostatin	n = 500 corpses with known time since death (between 1 day and 23 days)
Ortmann J. et al., 2017 [31]	Pancreas and thyroid	Insulin, glucagon, thyreoglobulin and calcitonin	n = 105 corpses with known time since death (between several hours and 22 days)
Mazzotti M.C. et al., 2019 [32]	Gingival tissue, sampled from the superior dental arch	Collagen type I and III	n = 10 corpses with known time since death (between 1 and 9 days)

## Data Availability

The data used to support the findings of this study are available on request from the corresponding author.
